# Preparation of high drug-loading celastrol nanosuspensions and their anti-breast cancer activities *in vitro* and *in vivo*

**DOI:** 10.1038/s41598-020-65773-9

**Published:** 2020-06-01

**Authors:** Tiantian Huang, Yian Wang, Yiping Shen, Hui Ao, Yifei Guo, Meihua Han, Xiangtao Wang

**Affiliations:** 1 Institute of Medicinal Plant Development, Chinese Academy of Medical Sciences, Peking Union Medical College, Beijing, 100193 China; 2 School of pharmacy, Henan University of Chinese Medicine, Zhengzhou, 450046 Henan China; 3 Center of Pharmaceutical Engineering Technology Research, College of Pharmacy, Harbin University of Commerce, Harbin, 150076 Heilongjiang China

**Keywords:** Drug delivery, Pharmaceutics, Breast cancer

## Abstract

As one of the main components of *Tripterygium wilfordii* Hook F, celastrol (CSL) has significant antitumor activity, but its clinical application has been limited by its poor solubility, low oral bioavailability and systemic toxicity. In this study, celastrol nanosuspensions (CSL-NSps) were prepared using an antisolvent precipitation method with poloxamer 188 (P-188) as a stabilizer at a high CSL/P-188 feeding ratio of 8:1. The resultant CSL was spherical in shape with an average particle size of 147.9 nm, a polydispersity index (PDI) of 0.12 and zeta potential of -19.2 mV. The encapsulation efficiency and drug loading content were 98.18% and 86.83%, respectively, and the X-ray diffraction (XRD) pattern showed that CSL existed in an amorphous state in the nanosuspensions. CSL-NSps were quite stable in various physiological media and plasma and were both suitable for oral and intravenous administration. Nanosuspensions greatly enhanced the *in vitro* dissolution, and the cumulative drug release reached approximately 69.20% within 48 h. *In vivo*, CSL-NSps (3 mg/kg, *i.g*.) displayed a significantly enhanced tumor inhibition rate (TIR) in comparison with that of CSL suspension when administered orally (TIR, 50.39%, vs. 41.16%, p < 0.05),  similar to that of PTX injection (8 mg/kg, *i.v*. TIR, 50.88%). CSL-NSps showed even better therapeutic efficacy than PTX injection (TIR, 64.18%, p < 0.01) when intravenously injected. This has demonstrated that, with the help of nanosuspensions, CSL is likely to be an effective and promising antitumor agent in clinic practice for the treatment of breast cancer.

## Introduction

In the past two decades, with the widespread application of high-throughput screening and combinatorial chemistry in drug discovery, many antitumor active compounds have been discovered, but most of that have shown poor aqueous solubility. This poor solubility is a major challenge for formulation of these compounds for both oral and intravenous administration^1,2^.

Celastrol (CSL), also named tripterine, is a pentacyclic triterpenoid extracted from the root bark of the traditional Chinese medicine *Tripterygium wilfordii* Hook F (TWHF). As the primary bioactive ingredient of TWHF, it exhibits a range of promising bioactivities, such as anti-inflammatory, anti-obesity, anti-diabetes, immunosuppressive, antioxidant, anti-Alzheimer′s disease, anti-Parkinson′s disease, anti-rheumatoid arthritis, anti-systemic lupus erythematosus, and anti-angiogenic effects^3–6^. In recent years, its anticancer effect has been widely recognized. It was proved that celastrol showed significant antitumor activity against lung cancer^7^, liver cancer^8^, gastric cancer^9^, breast cancer^10^, prostate cancer^11^ and cervical cancer^12^. CSL is a natural and multi-target antitumor drug. It can inhibit HSP90 and NF-κB signaling and activate the death signaling pathway of mitochondria, which ultimately induces tumor cell apoptosis^13^. Meanwhile, CSL is a P-gp inhibitor that can help overcome the emergence of multidrug resistance^14^.

However, CSL is a hydrophobic compound with low oral bioavailability and systemic toxicities^15^. Nanoparticle drug delivery system (NDDS), a nano drug delivery system with a particle size of 1–1000 nm formed by drugs and pharmaceutical materials, has shown good application prospects in the fields of targeted drug delivery and sustained-release drugs, improving the bioavailability of insoluble drugs and reducing side effects. Therefore, it has become a research hotspot in the field of pharmaceuticals^16^. In past years, CSL has been prepared into liposomes^17^, polymer micelles^18^, microemulsions^19^, phospholipid complex^20^ and solid lipid nanoparticles^21^, which has improved its solubility, absorption and bioavailability. But they has some limitations like drug loading, quality control difficulties, and scale-up difficulties.

Nanosuspensions are sub-micron colloidal dispersions composed of an almost pure drug core stabilized by a small amount of surfactants or amphipathic block-copolymers^22,23^. Therefore, nanosuspensions are characteristic of very high drug loading content, which reaches up to 100%. High drug loading content of the nanosuspensions could result in high drug delivery efficiency, effective cellular uptake and an adequate therapeutic drug concentration at the target site. Due to fewer pharmaceutical adjuvants used in the formulation, nanosuspensions can greatly reduce the risks and side effects related to pharmaceutical adjuvants, especially for intravenous administration. Similarly, nanosuspensions share the advantages of other NDDS, such as increased dissolution rate, elevated oral bioavailability, improved *in vivo* pharmacokinetics, passive targetability to solid tumors and inflammatory sites due to the enhanced permeability and retention (EPR) effect^24^. Thus, nanosuspensions have become one of the most promising drug delivery systems, especially from the viewpoint of drug economic value^25^. There are usually two main ways to make nanosuspensions: top-down and bottom-up. The latter one, represented by the antisolvent precipitation method, is widely used in laboratory research for its advantages, such as fewer drugs and stabilizers required for preparation, more choices for stabilizers, time-saving processes and no need for expensive equipment^26^.

In this study, CSL-NSps were prepared using P-188 as a stabilizer and an antisolvent precipitation method, which exhibited a high drug-loading content of approximately 86.83%. The physiochemical properties of the CSL-NSps and antitumor efficacy were evaluated. The results showed that CSL-NSps showed improved solubility and good antitumor activity in both oral and injectable formulations.

## Experimental Section

### Materials and instruments

Celastrol (CSL, purity >98%) was purchased from Aktin Chemicals, Inc. (Chengdu, China). Poloxamer 188 (P-188, 018K0029), Sodium oleate and 3-(4,5-dimethylthiazol-2-yl)-2, 5-diphenyltetrazolium bromide (MTT) were acquired from Sigma- Aldrich Co., (St Louis, MO,USA). Methoxy (polyethylene glycol) 2000–poly (e-caprolactone) 2000 (mPEG_2000_–PCL_2000_) was purchased from Jinan Daigang Biomaterial Co. Ltd. (Jinan, China). D-alpha tocopherol acid polyethylene glycol succinate (TPGS) was obtained from Xi’an Healthful Biotechnology Co. Ltd. (Xi’an, China). Albumin bovine V (>98%) (BSA) was acquired from Biotopped Co. Ltd. (Beijing, China). Paclitaxel (PTX) injections were purchased from the Beijing Union Pharmaceutical Factory (Beijing, China). Acetonitrile (HPLC grade) was supplied by Fisher Scientific (Pittsburgh, PA). The water used in the experiments was deionized.

### Cell lines and animals

The murine-derived breast cancer (4T1) cell line was obtained from Cell Culture Center, Institute of Basic Medical Sciences (Beijing, China) and grown in RPMI-1640 medium (RPMI 1640, Thermo Fisher Scientific), 10% fetal bovine serum (Thermo Fisher Scientific), 100 units mL^−1^ penicillin G and streptomycin with 5% CO_2_ atmosphere at 37 °C (Sanyo, Osaka, Japan).

BALB/c mice (18 ± 2 g body weight, 6 weeks of age) were purchased from Vital River Laboratory Animal Technology Co., Ltd. (Beijing, China) and were acclimated at a relative humidity of 70% ± 5% and 25 °C under 12 h light–dark cycle conditions for one week before the experiments.

All animal experiments were performed in line with the Guidelines and Policies for Ethical and Regulations for Animal Experiments and approved by the Institute of Medicinal Plant Development, China (Certificate number: 11401300078017, License Number: SCXK(Beijing) 2017-2020).

### Preparation of CSL-NSps

A simple antisolvent precipitation method was used to prepare CSL-NSps. Briefly, 8 mg of CSL powder was dissolved in 0.8 mL of ethanol as the organic phase, 8 mg of stabilizer was dissolved in 8 mL of deionized water as the aqueous phase, and the organic phase was then slowly dropped into the aqueous phase under the ultrasonic condition of 25 °C and 250 W (Ultrasonic cleaner, Kun Shan Ultrasonic Instruments Co., Ltd., China), followed by the removal of ethanol by vacuum rotary evaporation at 45 °C to obtain CSL-NSps. TPGS, mPEG_2000_-PCL_2000_, sodium oleate, and P-188 were used as stabilizers at a fixed CSL/stabilizer feeding ratio of 1:1. Then, CSL/P-188 at feeding ratios of 5:1 and 8:1 was used to prepare CSL-NSps.

### Physicochemical characterization of CSL-NSps

#### Dynamic laser light scattering (DLS) measurement

The mean particle size, PDI and zeta potential of CSL-NSps (diluted to approximately 1 mg/mL of the CSL equivalent concentration) were detected by a DLS method using a particle size analyzer (Zetasizer nano ZS, Malvern Instruments, UK). The measurement was performed at 25 °C, and each sample was measured in triplicate. All data were expressed as the mean ± standard deviation (SD).

### Morphology of CSL-NSps

The morphology of the CSL-NSps was observed via a JEM-1400 transmission electron microscope (TEM; JEOL, Tokyo, Japan). Briefly, 10 μL of water-diluted CSL-NSps (100 μg/mL) was dropped onto a 300-mesh copper grid, air-dried and dyed with 2% (w/v) uranyl acetate for 2 min. Then, the morphology of CSL-NSps was observed under TEM at an accelerating voltage of 120 kV.

### Powders X-ray diffraction (XRD) study

XRD patterns of CSL powder, P-188 powder, CSL-NSps lyophilized powder, and the physical mixture of CSL and P-188 powder were performed by an X-ray diffractometer (D8 Advance, Bruker, German) with Cu-Kα radiation generated at 40 kV and 100 mA. Samples were scanned over an angular range of 3°–80° of 2 θ, with a step size of 0.01°.

### Stability of CSL-NSps during storage

The CSL-NSps were kept at 4 °C and 25 °C, and the particle size and status of CSL-NSps were monitored at predetermined times.

### Stability of CSL-NSps in various physiological media and plasma

CSL-NSps were mixed with 1.8% NaCl, PBS (2×) and 10% glucose (1:1, v/v) or mixed with artificial gastric fluid, artificial intestinal fluid and rat plasma (1:4, v/v) and incubated at 37 °C^27,28^. The particle size was measured at specific time intervals. Each experiment was performed three times.

### Lyophilization of CSL-NSps

One mL of CSL-NSps was mixed with 15 mg of lyoprotectant (glucose, sucrose, whey protein and albumin bovine V), placed in an EP tube, covered with lens paper frozen for 6 h at −20 °C and lyophilized for 24 h in a vacuum freeze dryer (ALPHR 2–4 LD plus, CHRIST, Germany) under −43 °C, 0.12 mbar. The freeze-dried powder was dispersed in 1 mL deionized water, and the particle size and PDI value were measured by a particle size analyzer (Zetasizer nano ZS, Malvern Instruments, UK).

### Chromatographic condition for  CSL

The concentration of CSL was measured by high-performance liquid chromatography (HPLC, DIONEX, UltiMate 3000, Germany). A symmetry C18 column (4.6 mm × 250 mm, 5 μm, Venusil MP, Phenomenex) was used at 25 °C for chromatographic separation. The mobile phase was comprised of 85% acetonitrile to 15% water containing 0.005 M - phosphoric acid (v/v), with a flow rate of 1 mL/min. The detection UV wavelength was 425 nm, and the injection volume was 20 μL.

### Drug entrapment efficiency (EE) of CSL-NSps

The EE of CSL-NSps was determined by a centrifugal filtration method to distinguish the free drug from the heterogeneous system^29^. The CSL-NSps (0.5 mL) were placed in the inner chamber of a centrifuged filter tube (MWCO: 3,000) and then centrifuged against the filter for 20 min at 13,000 rpm. The drug concentrations in the filtrate (C_free_) and original nanosuspensions (C_total_) were measured by HPLC. EE is defined as the percentage of entrapped drug to total drug, which can be calculated by Eq. (1):

1 $${\rm{EE}}( \% )=({{\rm{C}}}_{{\rm{total}}}\mbox{--}{{\rm{C}}}_{{\rm{free}}})/{{\rm{C}}}_{{\rm{total}}}\times 100$$

### Drug loading content (DLC) of CSL-NSps

To measure DLC of CSL-NSps, the lyophilized CSL-NSps were weighed (W) and dissolved in methanol (V). The concentration of CSL was determined by HPLC (C). The DLC of CSL-NSps was calculated by Eq. (2):

2 $${\rm{DLC}}( \% )={\rm{C}}\times {\rm{V}}/{\rm{W}}\times 100$$

### *In vitro* drug release behavior

The CSL *in vitro* release behavior of the CSL-NSps was measured in a phosphate buffer solution (PBS, 0.01 M, pH 7.2-7.4). Briefly, CSL-NSps and free CSL (dispersed in 0.5% CMC-Na) were suspended in 1 mL of PBS solution with 0.5% Tween 80 and transferred into a dialysis bag (MWCO: 8,000 -14,000, Sigma). Then, the dialysis bag was placed in the PBS solution with 0.5% Tween 80 (50 mL) and shaken at 100 rpm at 37 °C. At each time interval, 1 mL of external media into which CSL was released was removed, and the same volume of fresh, original solution was added to each sample. The medium was renewed every 24 h. The concentration of released CSL was measured by HPLC. The above experiments were conducted in triplicate.

### Cytotoxicity assays

The cytotoxicity of CSL-NSps and free CSL was evaluated using an MTT assay on 4T1 cells. 4T1 cells were seeded into a 96-well plate at a density of 8 × 10^3^ cells/well in 0.15 mL of RPMI-1640 medium and cultured for 24 h. Subsequently, the cells were treated with different concentrations of CSL-NSps or free CSL (dissolved in DMSO, final concentration of DMSO < 0.1% diluted with a culture medium) for 48 h. Then, 20 μL of MTT solution (5 mg/mL) was added to each well and incubated for 4 h. Subsequently, the medium was substituted by 150 μL of DMSO and then shaken for 10 min. The absorbance values at 570 nm were measured using an ELISA plate reader (Biotek, USA). The cell inhibition rate was calculated according to the following equation:

3 $${\rm{Cell}}\,{\rm{inhibition}}\,{\rm{rate}}( \% )=(1\mbox{--}{{\rm{OD}}}_{{\rm{treated}}}/{{\rm{OD}}}_{{\rm{control}}})\times 100$$

where OD_treated_ was the absorbance measured from CSL-NSps or free CSL-treated wells, and OD_control_ was the absorbance measured from the blank culture medium-treated well.

The half maximal inhibitory concentration (IC_50_) value of CSL-NSps and free CSL was calculated using GraphPad Prism 5 (GraphPad Software, Inc., La Jolla, CA, USA).

### *In vivo* antitumor efficacy

4T1 cells (5.0 × 10^6^ cells/mL) was inoculated subcutaneously into the right armpit of 8 female BALB/c mice (0.2 mL each mouse). When the tumor volume reached approximately 1000 mm^3^, the mice were sacrificed by cervical dislocation, and the subcutaneous tumor tissue was collected and cut into pieces of 2 mm^3^. The vaccination site was sterilized routinely, and the tumor pieces were filled into the trocar needle and implanted subcutaneously into the right armpit within 30 min after the tumor tissue left the living body. After inoculation, BALB/c mice continued to eat and drink freely. When the tumor volume reached approximately 150 mm^3^, mice were randomly divided into six groups (n = 6) and were respectively dosed with normal saline (*i.v*., negative control group), PTX injection (8 mg/kg, *i.v*., positive control group,), P-188 solution (0.38 mg/kg, dissolved in normal saline, *i.v*.), CSL-NSps (3 mg/kg, *i.v*., test group 1), CSL-NSps (3 mg/kg, *i.g*., test group 2), or CSL suspension (3 mg/kg, *i.g*., test group 3). The CSL suspension was prepared by dispersing bulk CSL into deionized water containing 0.5% CMC-Na and sonicated for 5 min. The gavage groups were treated daily, and the tail vein injection group was treated every two days. All of the six groups were dosed continuously for 12 days, during which the tumor size and body weights were measured, and the tumor volume was calculated according to the following equation:

4 $${\rm{V}}=({\rm{a}}\times {{\rm{b}}}^{2})/2$$

where a and b respectively represent the length and width of the tumor. The tumor inhibition rate (TIR) was calculated using the formula:

5 $${\rm{T}}{\rm{I}}{\rm{R}}({\rm{ \% }})=(1-{{\rm{W}}}_{{\rm{t}}{\rm{r}}{\rm{e}}{\rm{a}}{\rm{t}}}/{{\rm{W}}}_{{\rm{c}}{\rm{o}}{\rm{n}}{\rm{t}}{\rm{r}}{\rm{o}}{\rm{l}}})\times 100$$

where W_control_ represents the average tumor weight of the negative control group, and W_treat_ represents the average tumor weight of the experimental groups.

### Statistical analysis

The data were presented as the mean values ± standard deviation (SD). Comparisons among the groups were calculated by one-way analysis of variance (ANOVA) using IBM SPSS statistics 23 software. Levels of *p < 0.05, **p < 0.01, and ***p < 0.001 were regarded to be statistically significant.

## Results and discussion

### Preparation and characterization CSL-NSps

Stabilizers are very important in the preparation of nanosuspensions, as they provide sufficient space resistance or electrostatic repulsion for the separate nanoparticles. The presence of excessive stabilizer in the nanosuspensions may result into the more solubilized drug and then accelerate the ripening effect. Otherwise, insufficient stabilizer could led to nanoparticle aggregation by bridging flocculation/aggregation, as the result of incomplete shielding of the nanoparticle surface. In this study, TPGS, mPEG_2000_-PCL_2000_, sodium oleate, and P-188 were used as stabilizers to prepare CSL-NSps with an initial feeding ratio of 1:1 (CSL: stabilizer, weight ratio). Since CSL showed good solubility in ethanol (over 20 mg/mL), and ethanol is cheap, safe and widely used in the pharmaceutical industry, ethanol was selected to be the organic phase for the preparation of CSL-NSps.

The particle size and PDI value of CSL-NSps prepared using different stabilizers are shown in Table 1. The data demonstrated that CSL is easy to fabricate into nanosuspensions (<200 nm) by the anti-solvent precipitation method without the need of further homogenization, whether TPGS, mPEG_2000_-PCL_2000_, sodium oleate, or P-188 was used as a stabilizer. Viewed from the PDI values alone, sodium oleate and P-188 were suitable stabilizers; judging from the storage stability alone, P-188 was the best stabilizer for CSL-NSps with similar mean particle size, PDI values and no aggregation for over 4 weeks of storage at 4 °C. CSL-NSps made from P-188 showed a mean particle size of 140.8 nm, PDI value of 0.14, and zeta potential of -18.8 mV. Meanwhile, P-188 is a cheap pharmaceutical adjuvant approved by the FDA for intravenous injection^30^. After comprehensive consideration, P-188 was selected as the best stabilizer to prepare CSL-NSps in the subsequent study.

**Table 1:** Particle size, PDI and potential of CSL-NSps prepraed using different stabilizers and at different drug-carrier ratio (n = 3).

Stabilizer	Drug: stabilizer	D_*h*_ (nm)	PDI	Zeta (mV)	Stability^a^	DLC %
TPGS	1:1	119.0 ± 2.46	0.20 ± 0.04	-9.44 ± 0.52	<4 weeks	^b^
mPEG2k-PCL2k	1:1	126.0 ± 1.94	0.23 ± 0.03	-10.2 ± 0.47	<4 week	^b^
Sodium oleate	1:1	181.1 ± 1.12	0.13 ± 0.02	-51.6 ± 0.35	<4 weeks	^b^
P-188	1:1	140.8 ± 1.17	0.14 ± 0.04	-18.8 ± 0.35	> 4 weeks	48.70
P-188	5:1	146.5 ± 3.16	0.11 ± 0.08	-19.9 ± 0.59	> 4 weeks	81.92
P-188	8:1	147.9 ± 2.16	0.12 ± 0.02	-19.2 ± 0.32	> 4 weeks	86.83

^a^Storage stability at 4 °C, meaning similar particle sizes and no aggregation;

^b^The data were not measured.

**Figure 1:**
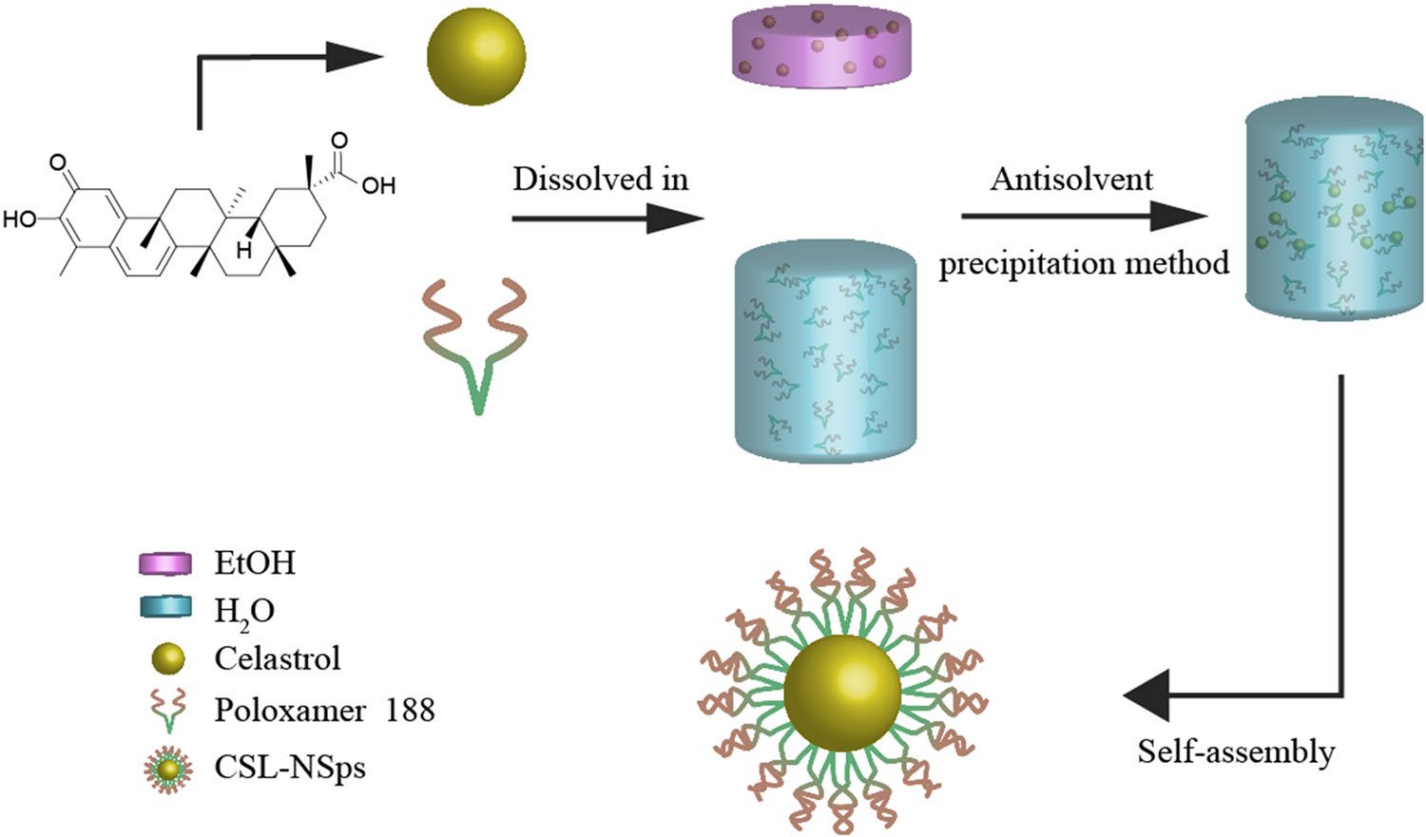
Schematic diagram of the preparation process of CSL-NSps.

**Figure 2:**
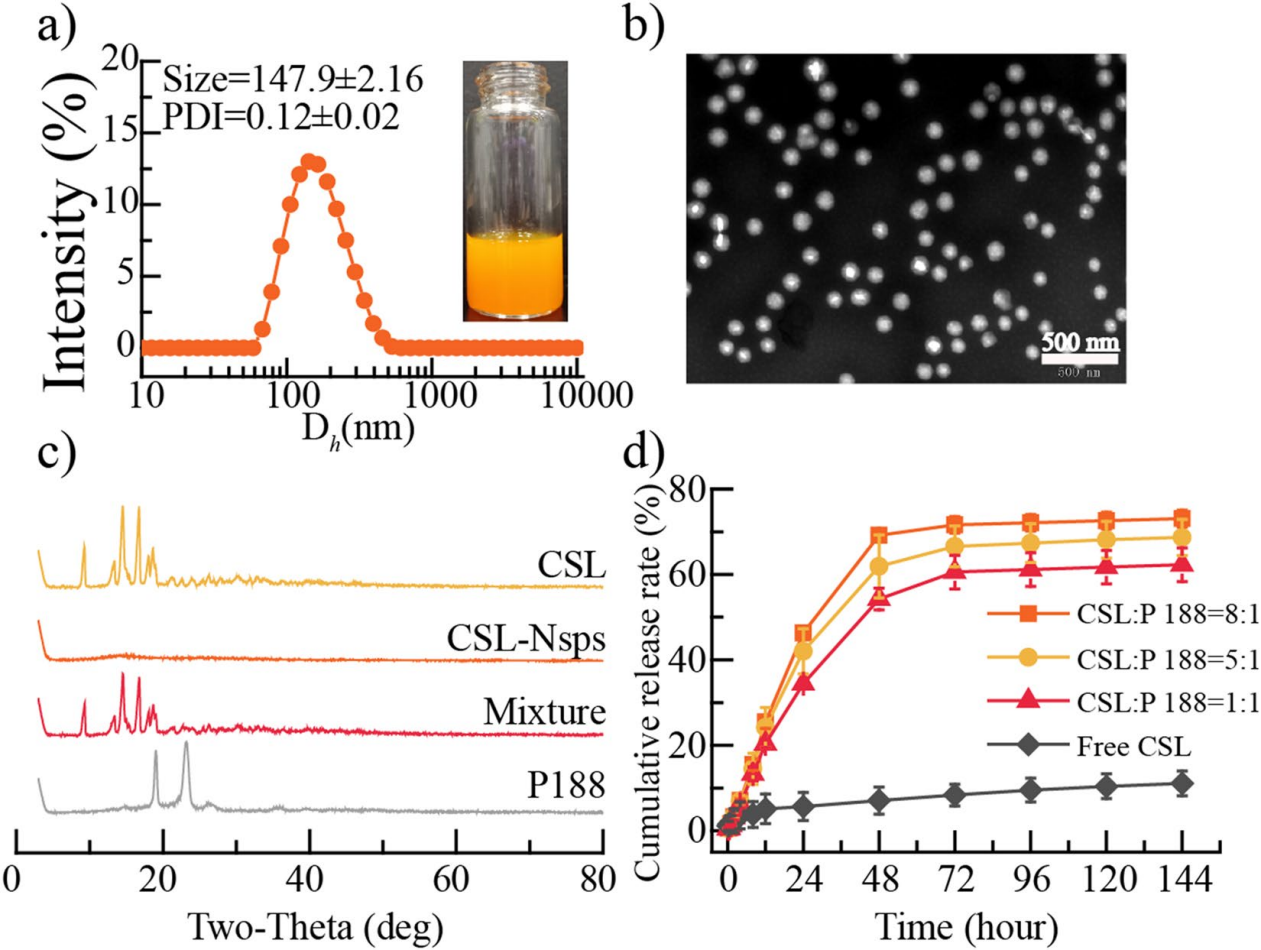
Preparation and characterization of CSL-NSps (**a)** The photograph and particle size distribution of CSL-NSps. (**b)** TEM photograph of CSL-NSps. **(c)** XRD pattern of the CSL, P-188, CSL-NSps, and the physical mixture of CSL and P-188. (**d)** *In vitro* drug release profiles of CSL-NSps and free CSL in PBS solution (pH 7.4) containing 0.5% Tween 80 at 37 °C (mean ± SD, n = 3).

Furthermore, higher CSL/P-188 feeding ratios (5:1 and 8:1) were also tried to prepare CSL-NSps (Table 1). The different CSL/P-188 feeding ratios (1:1, 5:1 and 8:1) made no difference in the particle size, PDI or zeta potential of the resultant CSL-NSps. In consideration of the high entrapment efficiency (98.18%), the high drug loading content and similar storage stability (Table 1), CSL/P188 nanosuspensions at a feeding ratio of 8:1 (CSL:P-188, weight ratio) were used in the subsequent experimental study in this paper. The self-assembly of CSL and P-188 into nanoparticles is illustrated in Fig. 1. The resultant CSL-NSps had a mean particle size of (147.9 ± 2.16) nm, with a small PDI of (0.12 ± 0.02) and a zeta potential of (– 19.2 ± 0.32) mV (Fig. 2a). The CSL-NSps were spherical, smooth and regular in shape by TEM (Fig. 2b). The particle size (< 100 nm) was less than that measured by DLS, as DLS gives the diameter of hydrated or “wet” nanoparticles, while TEM measures the size of dried nanoparticles. CSL-NSps exhibited a small size of <200 nm, which may be easily delivered to the tumor site via the EPR effect^31^.

The XRD patterns of the CSL powder, P-188, lyophilized CSL-NSps, and physical mixture of CSL and P-188 were measured under the same conditions (Fig. 2c). Both CSL and the physical mixture had sharp and intense diffraction peaks of CSL crystallinity in their diffractograms, which indicated that CSL existed primarily in crystalline in these two systems. However, no evident diffractogram peak was observed in the diffractogram of lyophilized CSL-NSps. This demonstrated that, during preparation of the CSL-NSps through antisolvent precipitation, the originally crystalline CSL had transformed into an amorphous state in the resultant CSL-NSps.

### Stability of the CSL-NSps

**Figure 3:**
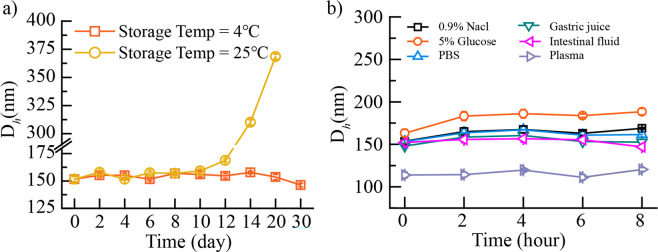
(**a**) Particle size change of CSL-NSps at 4 °C and 25 °C (mean ± SD, n = 3). (**b**) Particle size change of CSL-NSps after incubation with various physiological media at 37 °C (mean ± SD, n = 3).

The CSL-NSps were sealed and stored at 4 °C and 25 °C (Fig. 3a). One month of storage at 4 °C resulted in no significant change in the particle size of CSL-NSps. However, CSL-NSps could maintain stability in particle size only within 14 days when stored at 25 °C and then experienced a significant particle size increase in the following week, finally precipitating after one month. This indicated that the CSL-NSps are more suitable for storage at 4 °C.

To assess the suitability of CSL-NSps for intravenous injection and/or oral administration, CSL-NSps stability in the physiological media was investigated, by incubating CSL-NSps in physiological saline, PBS, 5% glucose, artificial gastric juice, intestinal fluid and plasma, and then measuring the particle size changes of the CSL-NSps in these by DLS at predetermined times (Fig. 3b). There was no significant particle size change and no aggregation observed for CSL-NSps in physiological saline, PBS, 5% glucose, artificial gastric juice, and intestinal fluid, indicating the high stability of the CSL-NSps in these physiological media and suitability of CSL-NSps for both intravenous injection and oral administration.

CSL-NSps showed a smaller particle size in plasma (about 115 nm) than in deionized water or PBS (about 150 nm), this is because that there are serum proteins and a small quantity of exosomes and other components in plasma, which made blank plasma as a whole look like a system containing tiny particles about 100 nm when measured using a DLS method. The particle size of CSL-NSps measured in this case was actually the average particle size of CSL-NSps themselves and the blank plasma. Similar phenomena have been observed and reported^32,33^. It is true that the absorption of serum protein on the surface of nanoparticles will increase the particle size of nanoparticles, but its contribution to the average particle size change depend on the surface property of nanoparticles and on the extent that nanoparticles absorb serum protein. In our study, it seems that the particle size reduction resulting from the presence of tiny particles in plasma significantly outweighed the particle size enlargement due to the protein absorption.

### Lyophilization

Colloidal system is a kinetically stable but thermodynamically unstable system, so CSL-NSps have to be lyophilized for long term of shelf storage. Nanoparticles tend to aggregate or grow in size by means of Ostwald ripening because of the very large surface area of nanosuspensions. Furthermore, nanosuspensions consisting of drug substances in the metastable amorphous form can undergo solid-state transformation, leading to crystal growth^34^. The presence of larger particles (in the micrometer range) is a safety concern because of the risk clogging of fine lung capillaries. Lyophilization (freeze-drying) or spray-drying can make the nanosuspensions minimize crystal growth during storage. Glucose, sucrose, BSA and whey protein were tried as lyoprotectants at a concentration of 1.5% (W/V) in this study. As shown in Table 2, lyoprotectants were indispensable for the lyophilization and the reconstitution of CSL-NSps. Glucose, sucrose, BSA and whey protein all displayed lyoprotective effects, among which BSA and whey protein were the best and showed excellent stabilization for CSL-NSps, with similar particle sizes and PDI values for the reconstituted CSL nanosuspensions to the original ones.

**Table 2:** Particle size and PDI of CSL-NSps before and after lyophilization.

Lyoprotectant	reconstitution	D_*h*_ (nm)	PDI
Before lyophilization	—	150.1 ± 2.32	0.17 ± 0.02
No lyoprotectant	precipitation	—	—
1.5% glucose	nanosuspension	792.0 ± 9.63	0.59 ± 0.05
1.5% sucrose	nanosuspension	268.3 ± 8.16	0.44 ± 0.06
1.5% whey protein	nanosuspension	151.2 ± 0.96	0.21 ± 0.02
1.5% BSA	nanosuspension	130.9 ± 1.53	0.17 ± 0.02

### *In vitro* drug-release behavior of CSL-NSps

The *in vitro* drug release profiles of CSL-NSps, whatever the CSL/P-188 feeding ratio (FR) was 1:1, 5:1 or 8:1, were represented as a two-phase pattern, a relatively fast drug release phase in the initial 48 h (first-order equation) with a cumulative drug release of approximately 54.26 ± 2.57% (FR, 1:1), 61.93 ± 7.38% (FR, 5:1) and 69.20 ± 0.63% (FR, 8:1) respectively, followed by a very slow drug release phase (Korsmeyer-Peppas equation) till 144 h, with a cumulative drug release reaching 62.29 ± 3.97% (FR, 1:1), 68.70 ± 4.25% (FR, 5:1) and 73.12 ± 1.96% (FR, 8:1) until the 144^th^ h (Fig. 2d). Since the different CSL/P-188 feeding ratios of (, w/w) shared the similar *in vitro* drug release pattern, it seemed the property of CSL, such as its solubility, played a major role in the release of CSL from CSL-NSps.

Although CSL-NSps prepared from the three feeding ratios released encapsulated CSL in the order of 8:1 > 5:1 > 1:1 in this study, but it doesn’t mean higher drug loading content always results in faster drug release. Relatively larger direct contact surface area of CSL-NSps with release medium and the subsequent relatively fast diffusion of CSL molecules from CSL-NSps, which benefited from the higher feeding ratio, may played a major role in this case. We have met the situation that different feeding ratios resulted into quite close *in vitro* release rate in the study of the nanosuspensions of other drugs (unpublished). And, in many polymeric micelles, lower drug-loading micelles had faster drug release.

Physical suspensions of CSL, made by dispersing CSL bulk powder in 0.5% CMC-Na were used as the control group and were treated under the same conditions. The cumulative drug release of CSL suspensions was only 11.09 ± 2.88% until 144 h,. indicating that nanosuspensions effectively enhanced the dissolution of CSL, which was mainly attributed to the small particle size, greatly enhanced surface area and the surface solubility of CSL by nanosuspensions. The amorphous state of celastrol in CSL-NSps was believed to be another reason, as the amorphous celastrol (high energy state) will be released faster than crystal celastrol (stable or low energy state, as in free celastrol suspensions) under the same condition.

### *In vitro* cytotoxicity assays

**Figure 4:**
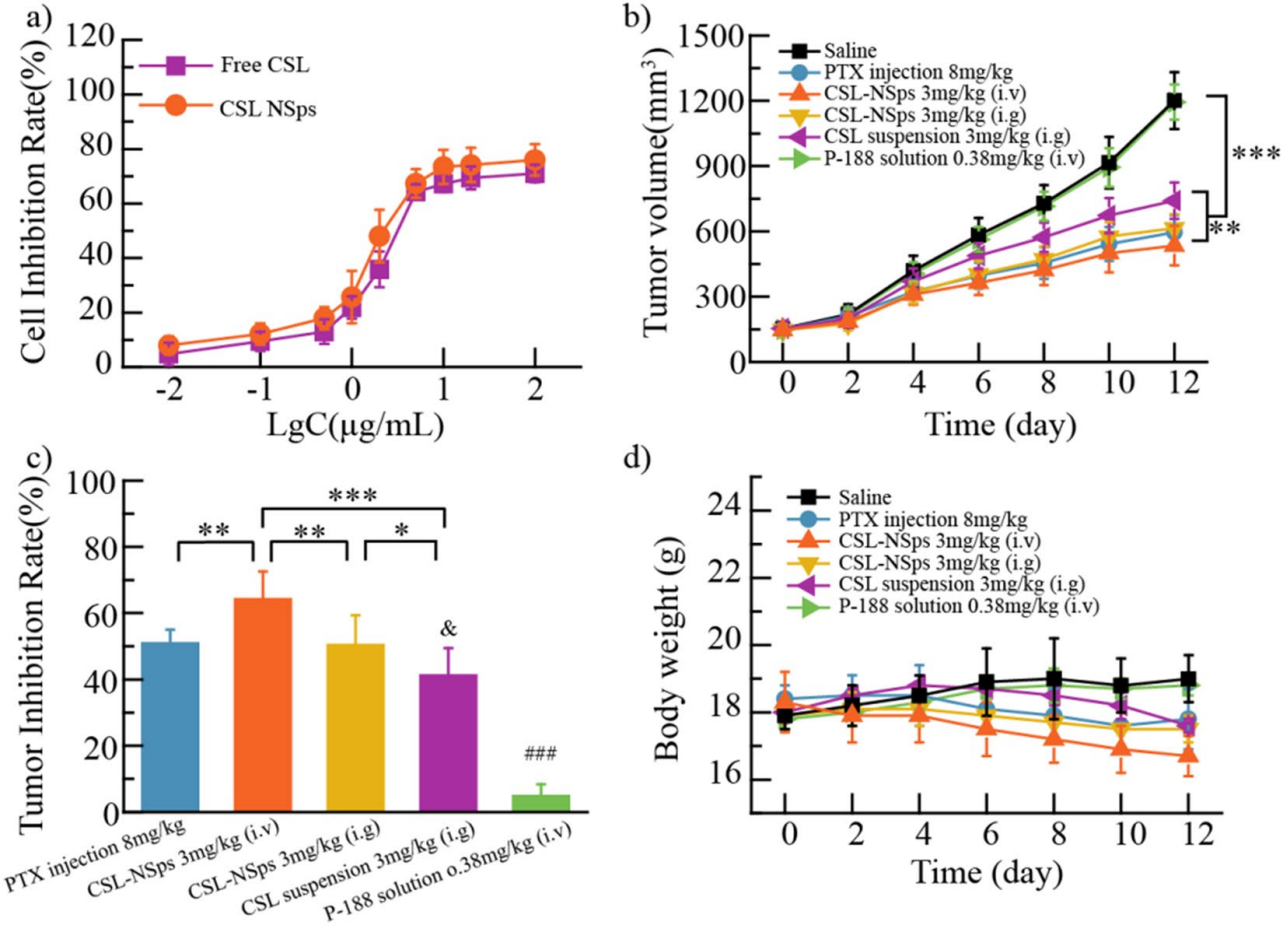
*In vitro* cytotoxicity and vivo antitumor efficacy: (**a**) Cytotoxicity of CSL-NSps and free CSL against 4T1 cells after 48 h of incubation (mean ± SD, n = 3). (**b**) Growth of tumor volume with administration in each group. (**c**) Inhibiting rate of CSL-NSps in 4T1 tumor-bearing mice. **(d**) Body weight change of mice upon administration (mean ± SD, n = 6, ^*^p < 0.05 vs. CSL-NSps *i.g*, ^**^p < 0.01 vs. CSL-NSps *i.g*. and PTX group, ^***^p < 0.001 vs. CSL suspension, ^&^p < 0.05 vs. PTX group, ^###^p < 0.001 vs. other group).

4T1 cells were incubated with different concentration of CSL-NSps and free CSL for 48 h to explore their cytotoxicity via an MTT assay (Fig. 4a). In contrast to free CSL, with an IC_50_ value of 4.11 μg/mL, CSL-NSps displayed slightly enhanced inhibitory effect against 4T1 cells, with an IC_50_ value of 2.82 μg/mL (p > 0.05). The enhanced antitumor efficacy of nanoparticles could be explained by the fact that tumor cells can conduct nonspecific adsorption to drug-loading nanoparticles and enhance their internalization via endocytosis and other mechanisms^35^. In contrast, the free CSL crossed the cell membrane via passive diffusion.

### *In vivo* antitumor efficacy

The *in vivo* antitumor efficacy of CSL-NSps was conducted on 4T1 tumor-bearing mice. Raw data of body weight, tumor volume and TIR calculations are available in the Supplementary Dataset. As shown in Fig. 4b, at the end of the experiment, the tumor volumes were increased by 8.0-fold for saline and P-188 solution, 4.0-fold for PTX injections, 3.7-fold for CSL-NSps (*i.v*.), 4.2-fold for CSL-NSps (*i.g*.) and 4.8-fold for CSL suspension. CSL-NSps (*i.v*.) showed highest antitumor efficacy, significantly better than CSL suspension (p < 0.001), followed by PTX injections (p < 0.05 vs. CSL suspension). Blank vehicle (P-188 solution, 0.38 mg/kg, *i.v*., equivalent to the dose of P-188 in CSL-NSps) displayed little inhibition against the *in vivo* tumor growth (p > 0.05 vs. normal saline group).

The tumor inhibition rate (TIR) calculated by the average tumor weights are shown in Fig. 4c. Although daily oral administration of CSL suspension showed a TIR of 41.16%, nanosuspensions successfully improved its TIR to 50.39% (p < 0.05) at the same dose and through the same administration route, quite close to that of PTX injections (50.88%, p > 0.05), indicating the good prospect of CSL-NSps in the tumor treatment. This result also certificated that nanosuspensions evidently improved the oral bioavailability of CSL. Intravenous injection further elevated the therapeutic efficacy of CSL-NSps, achieving a TIR of 64.18% (p < 0.01, vs. *i.g*.), significantly higher than that of PTX injection (p < 0.01) (Fig. 4c).

Body weight changes in the mice during the treatment course were also monitored to evaluate the systematic toxicity of the drug formulations. As shown in Fig. 4d, saline group and P-188 solution group displayed very limited body weight increase, while PTX group, CSL-NSps groups and CSL suspension all occurred body weight losses, indicating that CSL had little systemic toxicity.

Furthermore, our research also has some limitations. For instance, there is no in-depth study on the mechanism of nanoparticle induced tumor cell apoptosis and *in vivo* pharmacokinetics study, which will be the focus of further research.

## Conclusions

The discovery of new antitumor agents and their effective *in vivo* delivery are still important tasks owing to the wide existence and recurrence of multidrug resistance of antitumor drugs used in the clinic. In this study, CSL-NSps were prepared successfully, with a particle size of 147.9 nm, a narrow size distribution and an excellent EE and DLC of 98.18% and 86.83%, respectively, by the antisolvent precipitation method. CSL-NSps showed clear stability in various physiological media and a continuous release for 144 h *in vitro*. *In vitro* cytotoxicity experiments showed that CSL-NSps have a smaller IC_50_ value than free CSL for 4T1 cells (2.82 μg/mL vs. 4.11 μg/mL), but the IC_50_ values of the two groups were not significantly different. *In vivo* experiments demonstrated that the oral administration of CSL-NSps produced higher antitumor activity than the CSL suspension (p < 0.05) and similar antitumor activity as the PTX injections. Intravenous injection of CSL-NSps (3 mg/kg) demonstrated higher antitumor activity than the PTX injections (8 mg/kg) (p < 0.01). CSL-NSps may be a potent antitumor drug and can be used in the clinic due to its excellent antitumor effects.

## Supplementary information

Dataset 1.
